# Common Sense

**Published:** 1880-02

**Authors:** 


### HALL'S JOURNAL OF HEALTH.

OUR LEGITIMATE SCOPE IS ALMOST BOUNDLESS: FOR WHATEVER BEGETS PLEASURABLE AND HARMLESS FEELINGS, PROMOTES HEALTH; AND WHATEVER INDUCES DISAGREEABLE SENSATIONS, ENGENDERS DISEASE.

### COMMON SENSE

Is not the practical sense of the great mass of the people, it is rather the legitimate deduction drawn by a wise man from any given premises; it is the instinctive course pursued when action is required by a mind working with perfect freedom in the light of truth. Scarce a man who reads this article but has found a dozen times, that in washing his hands with his coat off, the wristbands of his shirt, if unbuttoned, will slide down towards the hands and be dribbled with water. And yet the very next time the hands are washed under the same circumstances, the wristbands will be pushed up, in the hope that they will remain up. In this case laziness prevents the mind from working freely, from looking at the facts of the case, and drawing natural inferences. The arm forms toward the wrists an inclined plane, made more inclined by the fact that in washing, the fingers are lower than the wrists below a horizontal line. Common sense dictates that the wristbands left unsupported could only maintain their place under the circumstances by the annihilation of the great first law of matter, gravity. A cotemporary says on the general subject,

"If common sense were an article to be bought in the market, doubtless there would be a great demand for it; or if not, it would be well for the corporation to make an appropriation of the public moneys to buy up a lot, from which the needy might draw without any charge. It is about as essential as Croton water to our daily comfort, but there are a great many elegant looking houses into which it has not yet been introduced. The very low-born, the totally ignorant, who find it difficult to distinguish between the suggestions of conscience, the promptings of common sense, and the false light of superstition, which they mistake for knowledge, are only pitiable. But those who were born to an inheritance of common sense, and have wasted it, deserve our reprobation and contempt.

"If half the sensible people in the world had common sense, it would be better; but, unfortunately, most men's judgments slide in between their prejudices and their education, like windows in badly-fitting sashes; when you attempt to bring them to the position they were made to take, they give first on this side and then on that, and particularly happy you may feel yourself if you can bring them into position without putting out a light."

In reference to health, common sense teaches that every time a man puts his finger in the fire it will be burned; that if a certain article of food eaten to-day gives discomfort, it will do it to-morrow; that if a hearty and late supper prevents sound refreshing sleep one night, it will do it another; that if exposure to a draft of air while perspiring gives a bad cold once, it will do it again; yet there are multitudes who get old before they learn to heed these things, to have "common sense."

"The human body is a very delicately-constructed machine. Yet as the City Hall clock, which every body pronounces an excellent one, took the liberty to stop, a few days since, when a boy pushed his chair up against the 'compensator;' so the human mechanism will not move true and steady, if ignorant men are allowed to play with 'the works.' Seeing that there is not room for the finest cambric needle to lie, without producing mischief, any where within the several solid feet that constitute the body of a man, common sense would satisfy an appreciative person that he cannot accommodate within his living tissues a pound of drugs, every grain of which penetrates farther than needles and blocks, or throws off the track, the wheels of every rolling globule of blood in his veins.

"Common sense takes the stump, and labors to convince sensible people that when they are sick the thousandth part of a grain of any material, of which they have taken a drachm since dinner, and been neither better nor worse therefor, cannot materially modify their condition. Yet men who are good for making money, and who do not educate their children with specific reference to making fools of them, are stone deaf on the side that common sense whispers his admonitions; spend goodly sums on the quack who indulges them in the luxury of being cheated, and enjoy the high satisfaction of being wonderfully cured where nothing under the sun has ailed them. Common sense, of course, shakes off the dust of his feet, and leaves to his fate one whose phrenological developments would justify the suspicion of a moderate share of intelligence, when he makes phrenology ridiculous, and belies all the indications of physiognomy by imbibing bottle after bottle of Nervous Antidote, Cherry Bitters, Choice Catholicons or Renovating Resolvents, to cure ailments whose characters differ in every respect from each other; just as if all diseases were like the vermin of all sorts that haunt old alms-house cellers, and all alike were best disposed of by being drowned out of their quarters.

"But it seems to us as if common sense were particularly ashamed of those stout, stalwart bodies, in which strong minds, like engines of many horse-power, were originally set up, when, instead of trusting to their own powers, and heeding their own capacities, they give themselves up to the guidance of other men, in matters which they ought thoroughly to understand for themselves. When a good skipper is going through Hurl-Gate, he does very well to ask a pilot on board if he does not know the rocks; but when he is fairly out on the Sound, with a fair wind and a clear night, when the compass is a good one, and he knows all the lights from Sandy Point to Little Gull, he is weak and wasteful to be at the expense of a pilot's fees. So when a man is sailing among colics and pains of any sort, of which he does not know the nature, he cannot do better than order on board a skillful physician, who has sounded every foot of the way, and knows when to give a fuller sheet, when to haul close, and when to put the craft square before the wind, and trust every thing to his care, till the ripples are all past and the waves chase each other, without any sudden breaks or declension, to right or left, as if a rock were just below. But for a full-grown man, who is well, to call in a doctor to know if he may eat this delicious fruit or that, may make this pleasure trip or that, may tarry within the bounds of the city till his business will permit his removing to the country, or must push at once into summer quarters, it is simply ridiculous, and common sense objects to being claimed by him as an acquaintance.

"*Ten o'clock.*—But you, my dear fellow, ought to be a-bed. Have not you read ALCOTT, and GRAHAM, and FRANKLIN, and SINCLAIR? Haven't you studied Hygiene, and attended a course of popular lectures on the subject? Haven't you studied the rules of longevity? Don't you know that every hour less than seven of sleep at night, is a day deducted from the sum total of your life? and that, from Dr. JOHNSON to TODD's Student's Manual, all the authorities agree that an hour of sleep before midnight is worth two after it?

"No! no! don't go to writing now. Don't raise the steam at this time of night. You would not let your housekeeper begin her baking now, neither should you set your brain to seething so unseasonably. It was a wise man—and a little time spent among our books would enable us to give his name—who allowed no serious book to engross his attention after his evening meal, and indulged himself in no severer labor than a game of romps with his children."

